# The Effects of Storage Time and Environmental Storage Conditions on Flour Quality, Dough Rheology, and Biscuit Characteristics: The Case Study of a Traditional Italian Biscuit (Biscotto di Prato)

**DOI:** 10.3390/foods11020209

**Published:** 2022-01-12

**Authors:** Alessio Cappelli, Andrea Bini, Enrico Cini

**Affiliations:** Department of Agriculture, Food, Environment and Forestry (DAGRI), University of Florence, Piazzale delle Cascine 16, 50144 Florence, Italy; andrea.bini3@stud.unifi.it (A.B.); enrico.cini@unifi.it (E.C.)

**Keywords:** warehouse temperature, warehouse moisture, starch, protein, gluten, alveograph parameters, flour moisture

## Abstract

Many types of baked goods are firmly rooted in the food habits of many people in different countries. Although there have been great strides in improving milling, kneading, and baking, given the lack of essential studies, further steps forward need to be taken to understand the effects of storage time and environmental storage conditions, thus motivating this work. The aim of this study is to assess the effects of storage time, using one-way ANOVA, and environmental storage conditions (environmental temperature and humidity), using MOLS analysis, on flour composition, dough rheology, and biscuit characteristics. Seven levels of storage time were tested: T0 (control), T1, T2, T3, T4, T5, and T6. The results showed that flour storage time significantly increased dough tenacity (P) and curve configuration ratio (P/L), and decreased the biscuit volume (best at T0). However, 2–3 weeks of storage highlighted a significant increase in deformation energy (W), an essential alveograph parameter that is closely correlated to the technological success of leavened products. This optimum found for W might be considered as a great stride in understanding the effects of storage time, confirming that wheat flour can reach its optimal performance after two-three weeks of storage, in particular for W. Moreover, this information could be useful, not only for biscuits production, but also for bread and bakery products (and, thus, the entire bakery industry). MOLS analysis highlighted that dough rheology and biscuit characteristics are mainly affected by flour composition (primarily from starch content) rather than environmental storage parameters. In conclusion, to optimize the biscuit characteristics, it is necessary to use flours with a low content of damaged starch by selecting the most suitable milling technique and carefully managing the operative parameters.

## 1. Introduction

Bread and bakery products are considered worldwide as staple foods, essential for human nutrition. As highlighted in an earlier work [1], bread, in particular, can be considered as the oldest type of baked good (dating back to 10,000 years BCE). However, nowadays, many types of baked goods are firmly rooted in the food habits of many people in different countries [2]. As a result, several types of bakery products can be produced in different countries, regions, or, for example in Italy, in different municipalities [1].

One example might be a traditional Tuscan biscuit named biscotto di Prato. This traditional product takes its name from the municipality of production (i.e., Prato), a town very close to Florence. However, it is very important not to confuse the biscotto di Prato with the well-known Cantuccini Toscani PGI (with almonds). In fact, although these biscuits look similar at first glance, they have some important differences. Firstly, Cantuccini Toscani have the PGI denomination, unlike biscotto di Prato. Secondly, these biscuits are produced following different ingredients and recipes (Cantuccini Toscani PGI are produced using wheat flour, sugar, butter, honey, eggs, almonds, leavening agent (ammonium bicarbonate or others), and salt, while biscotto di Prato is produced using only wheat flour, sugar, eggs, almonds and pine nuts). However, despite the different recipes could influence the final products characteristics, as highlighted in earlier works, these products are mainly influenced by the four major stages of the production chain, i.e., milling, kneading, leavening, and baking [1,3,4,5,6,7,8,9,10].

With respect to the milling process, Cappelli et al. (2020) [6] summarized current knowledge regarding stone and roller milling. The findings highlighted that the selected milling technique had the highest impact on flour quality, dough rheology, and final bread characteristics [6]. To improve stone milling, the most interesting strategies are focused on the correct management of wheat conditioning and stone rotational speed [5], as well as the modernization of traditional stone watermills [11]. On the other hand, to improve roller milling, other strategies have been suggested. These include improving automatic and adaptive mill plants [12]; the use of the break, sizing, and reduction systems of the roller mill for flour differentiation, improving milling technology, flour quality, and reducing environmental impacts [13]; and, finally, the grain debranning before milling, combined with the stabilization of bran, middlings, and germ, for the extension of whole wheat flour shelf life [14,15].

Although milling has the greatest impact on dough rheology and bread characteristics, the kneading process also plays a key role [7,8]. Cappelli and Cini (2021) [4] highlighted that it is essential to correctly manage kneading time, dough temperature, mixing speed, dough aeration, and other key parameters, to guarantee optimal dough rheology and best bread characteristics. Regarding the improvement of the kneading process, control the dough temperature during kneading using alternative, eco-sustainable, refrigerants (like carbonic snow) [7,9,16], correctly manage the water addition during kneading [8], delay the addition of bran and middlings during kneading in whole wheat dough and bread production [17], and, finally, develop automatic, adaptive, and more usable kneading machines [8,18,19,20], seem to be the most interesting strategies.

Subsequently to kneading, another important unit operation, namely leavening, can significantly affect the bread and bakery products characteristics. As highlighted by Venturi et al. (2021) [10], the selected leavening agent could significantly affect the technological and nutritional characteristics of the baked goods. In particular, the latter authors assessed the effects of baker’s yeast, biga, and sourdough on bread characteristics. The results showed a marked effect of the leavening agent on final bread characteristics [10]. In particular, the use of sourdough in ancient wheat flour breadmaking had a sharp effect, levelling the potential differences due to the flour, leading to bread with similar characteristics to the loaves produced using modern cultivar flours [10]. However, Cappelli et al. (2021) [1] highlighted the importance of selecting the optimal baking technique as a function of the desired product characteristics. The latter authors found that conventional ovens could be improved through combinations with steam and vacuum technologies. Moreover, microwave baking could also be significantly improved by hybridization with other baking techniques (in particular with IR and IR-visible heating) [1].

Although scientific and technological research has made great strides in improving the four major stages of the production chain, given the lack of essential studies, further steps forward need to be taken to understand the effects of storage time and environmental storage conditions, thus motivating this work. In particular, only a few papers regarding the effects of flour storage in controlled conditions for bread production are available in the literature. Moreover, specific papers assessing the effects of flour storage in uncontrolled environment (typical of many food companies) on flour quality, dough rheology, and biscuit characteristics are lacking, highlighting the novelty of this research. As a result, the aim of this study is to assess the effects of storage time and environmental storage conditions (i.e., environmental temperature and humidity in the company warehouse), on flour composition, dough rheological properties, and biscuit characteristics.

## 2. Materials and Methods

### 2.1. Raw Materials

**Figure 1:**
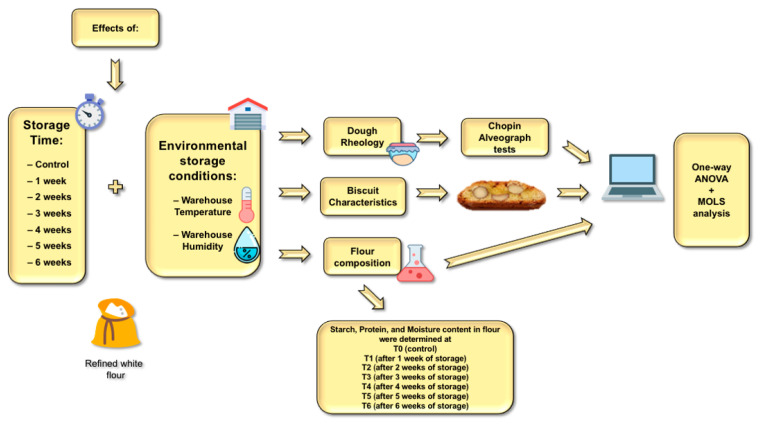
Graphical representation of the experiment.

Commercial Italian wheat flour (*Triticum aestivum*), defined 00 under Italian legislation (ash content ≤ 0.55%, protein content ≥ 9.00%), white sugar (100% sucrose), almonds, and pine nuts were kindly provided by Biscottificio Antonio Mattei Ltd. (Prato, Italy). The tested flour, produced from the same batch of wheat, was provided by Molino Cerioli (Parma, Italy). Immediately after milling, the flour was sent to Biscottificio Antonio Mattei Ltd. to implement the experimental trials. As soon as the flour was received, the first control sample (i.e., T0, which refer to zero weeks of storage) was used to carry out flour characterization analyses, dough rheological assessment, and biscuit production. Successively, flour was stored in the warehouse of the company using 25-kg paper bags divided into six different pallets (corresponding to the six storage time tested in this work (T1 (one week), T2 (two weeks), T3 (three weeks), T4 (four weeks), T5 (five weeks), and T6 (six weeks))). The aforementioned raw materials were used for the production of the tested biscuits (biscotto di Prato), following the Biscottificio Antonio Mattei traditional recipe and using the machines and plants of the factory located in Prato. A graphical representation of the experiment is reported in Figure 1.

### 2.2. Environmental Storage Conditions Measurement

Environmental temperature and humidity in the company warehouse were measured using a thermo-hygrometer (Bresser, model 7000020CM3000) able to detect temperature and humidity variations in the company’s warehouse during the trials. The thermo-hygrometer was placed next to the flour pallets to have a reliable measure of temperature and humidity. Moreover, these parameters were recorded every five hours (6:00 a.m., 11:00 a.m., and 4:00 p.m. (company closing time)), three days a week (Tuesday, Thursday, and Saturday) for seven weeks (from 27 April 2021 to 12 June 2021).

### 2.3. Flour Characterization

Flours analysis was carried out by the Analytical Food Laboratory (Florence, Italy), according to AOAC approved methods [21]. In particular, protein (AOAC 920.87 [21]), starch (AOAC 979.10 [21]), and moisture (AOAC 925.10 [21]) content were determined following approved, official methods. All tests were carried out in three replicates.

### 2.4. Rheological Properties

Rheological properties were evaluated using a Chopin NG alveograph, linked to an alveolink integrator–recorder (Chopin technologies, Villeneuve-La-Garenne, France). Consistently with the procedure described in ISO 27971 [22], dough tenacity (P), dough extensibility (L), deformation energy (W), the index of swelling (G), and the curve configuration ratio (P/L) were evaluated. All tests were carried out in three replicates.

### 2.5. Biscuit Production Process and Characterization

The production process of the tested biscuits (biscotto di Prato), followed the Biscottificio Antonio Mattei traditional recipe using the machines and plants of the factory located in Prato (Italy). The biscuits production was carried out every Tuesday, for seven weeks (from 27 April 2021 to 12 June 2021), keeping the same recipe. Moreover, all tests were carried out in three replicates. Following a randomized scheme developed using the statistical software R, from three different bags randomly selected, 7 kg of flour were sampled and placed in a container. Successively, the 21 kg of flour was homogenized, and a representative sample was collected and sent to the Analytical Food laboratory for flour characterization, as reported in Section 2.3. Successively, the homogenized flour was kneaded with the others ingredients reported in Section 2.1, using an industrial double-arm mixer (model ITF 80, Pietroberto, (Vicenza, Italy)), to produce the biscuits dough. 

Shortly afterwards, the dough divider machine helped to split the mass in several smaller doughs which were subsequently formed using the molder machine. Successively, the small loaves of dough were baked at 230 °C for 18 min. At the end of baking, following a randomized scheme developed using the statistical software R, three baked small loaves were selected to obtain the tested biscuits. In particular, a single biscuit was collected exactly from the center of each small loaf. After one hour cooling, the biscuit volume (L) and height (mm) were measured. The standard millet displacement method [23] was used to measure biscuit volume (L), consistently with an earlier work [17]. Moreover, the biscuit height (mm) was measured with a caliper at the center of the biscuit. All tests were carried out in three replicates.

### 2.6. Statistical Analysis 

The effects of storage time were assessed using a one-way ANOVA, which was applied to each alveographic and biscuit parameter to establish its significance. Seven levels were tested: T0 (control), T1 (one week of storage), T2 (two weeks of storage), T3 (three weeks of storage), T4 (four weeks of storage), T5 (five weeks of storage), and T6 (six weeks of storage). Significance was set at *p* < 0.05. In cases of statistically significant results (*p* < 0.05), a Tukey HSD post hoc test was performed.

The effects of environmental storage conditions, specifically environmental temperature and humidity in the company warehouse, were investigated with a multiple ordinary least square regression (MOLS). MOLS allowed to assess the relationship between rheological properties (i.e., alveograph parameters G, P, L, W, and P/L) and biscuit parameters (volume and height) with flour components (moisture, starch, and protein) and environmental storage conditions (i.e., mean temperature and humidity of the week) through the development of specific models. The aim was to determine, according to the R^2^ of these models, which factor between storage temperature, storage humidity, and flour composition has determined the most significant effects on dough rheology and biscotto di Prato characteristics. 

In particular, the following models were investigated: (1) single alveographic parameters (G, P, L, W, and P/L) versus the compositional parameters of the flour (moisture, starch, and protein); (2) single alveographic parameters (G, P, L, W, and P/L) versus mean temperature and humidity of the week; (3) Biscotto di Prato volume and height versus the compositional parameters of the flour (moisture, starch, and protein); (4) Biscotto di Prato volume and height versus mean temperature and humidity of the week. The statistical analysis was carried out using R software.

## 3. Results and Discussion

### 3.1. Results of Flours Characterization and Environmental Storage Conditions 

The results of the flours characterization are reported in Table 1.

Flour moisture significantly increases in the first week of storage and then slowly decreases till the last week of storage. In particular, samples T1, T2, T3, and T4 showed a significant increase in moisture content. The highest flour moisture in sample T1 might be due to the highest environmental humidity in the first week of storage (week 0), as shown in Table 2.

Successively, the environmental humidity in the company warehouse decreases leading to a decreasing trend in flour moisture from sample T2 to sample T6. With respect to starch content, the one-way ANOVA found that sample T0 had a significant lower starch content compared to the other samples. Moreover, the results reported in Table 1 show a slightly increasing trend as the storage time increases. As highlighted by Lancelot et al. (2021) [24], this is probably due to the activity of α-amylase which can slightly increase the damaged starch content in flours during storage. Regarding the protein content, the tested flours showed different values (Table 1). Contrarily to starch, the protein content in sample T0 is significantly higher compared to the other samples. However, as highlighted in Table 1, no clear trend is discernible from the results, highlighting a protein variability mainly related to the milling process and to other parameters associated to the flour supplier (although the flour was obtained from the same batch of wheat).

The results of temperature and humidity evolution in the warehouse of the company during flour storage are reported in Table 2. As expected, temperature in the company warehouse increases progressively from week 0 to week 6 (Table 2). On the contrary, humidity decreases from week 0 to week 5, with the exception of week 6 where a slight increase was found (probably due to rainy days). However, the environmental storage parameters (i.e., temperature and humidity) show expected trends since, in the tested weeks (from 27 April 2021 to 12 June 2021), spring was gradually making room to summer. 

### 3.2. Dough Rheological Properties

With respect to the effects of storage time on dough rheological properties, Table 3 summarizes the results of the Chopin alveograph tests.

Regarding dough tenacity (P), the one-way ANOVA found a significant main effect for the factor storage time (*p* < 0.001). As shown in Table 3, the P value of the sample T0 is significantly lower compared to all the other samples, highlighting an increase in P during storage, consistent with Lancelot et al. (2021) [24] and González-Torralba et al. (2013) [25]. Moreover, MOLS analysis identified a statistically significant relationship between P and starch (*p* = 0.044) with a high amount of variance (R^2^) explained by this model (i.e., R^2^ = 0.81), consistently with earlier works [26,27].

As highlighted by Cappelli et al. (2018) [26] and Mastromatteo et al. (2013) [28], the increase in P during storage seems to be more related to starch content rather than to environmental storage conditions. In particular, samples T1, T2, T3, T4, T5, and T6 have a higher starch content compared to T0, which have the lowest P value. The higher starch content in these samples increased the water-holding capacity, leading to an increase in viscosity and strength in the tested doughs, with subsequent increase in P [26,28]. Moreover, MOLS analysis found a statistically significant relationship between P and weekly mean humidity (*p* = 0.006), consistent with González-Torralba et al. (2013) [25], but with a lower amount of variance explained by the model (i.e., R^2^ = 0.35). In conclusion, despite González-Torralba et al. (2013) [25] found that high humidity can increase dough tenacity (P) and strength (W), changes in P seem to be mainly related to flour composition (in particular to starch content).

With respect to L and G, the one-way ANOVA did not find any statistically significant differences. However, MOLS analysis identified a statistically significant relationship between L and protein (*p* = 0.027) and between G and protein (*p* = 0.031) with a low amount of variance explained by these models (i.e., R^2^ = 0.36 and 0.37, respectively). Despite the low variance explained by these models, the protein content significantly influence L and G. As reported in Table 3, the sample T0 had the highest protein content and this probably led to the highest L and G values [29,30]. This is supported by Wang et al. (2002) [31] who found that wheat storage proteins (gliadin and glutenin) mainly influence the extensibility of doughs. Finally, MOLS analysis did not find any statistically significant relationship with the environmental storage parameters (i.e., weekly mean temperature and humidity).

Concerning W, the one-way ANOVA found a significant main effect for the factor storage time (*p* = 0.002). In particular the samples T2 and T3 had significantly higher W values compared to the other samples (Table 3). This is consistent with Quaglia (1984) [32], who highlighted that wheat flour reaches its optimum after two-three weeks of storage, in particular for W. Moreover, MOLS analysis identified a statistically significant relationship between W and flour moisture (*p* = 0.001), protein (*p* = 0.020), and starch (*p* = 0.025), with a good amount of variance explained by the model (R^2^ = 0.57). Regarding flour moisture, the samples T2 and T3 had the same value (15.10), highlighting an optimum for W at this specific flour moisture content (Table 2). Moreover, as highlighted in earlier works [26,27], W is closely correlated with protein and starch. In particular, higher starch content is related to an increase in dough viscosity and strength, due to increased water-holding capacity, which led to higher W values [26,27,28]. Moreover, an optimal balance between protein and starch content in wheat flours could improve W values, as found for samples T2 and T3 [33]. Finally, MOLS analysis did not find any statistically significant relationship with the environmental storage parameters.

With regard to P/L, the one-way ANOVA found a significant main effect for the factor storage time (*p* = 0.002). As shown in Table 3, the sample T0 shows the best P/L value, which is significantly lower compared to all the other samples. The increase in P/L during storage is consistent with Lancelot et al. (2021) [24] and González-Torralba et al. (2013) [25], which highlighted similar trends mainly due to the increase in P and the decrease in L during flour storage. Moreover, MOLS analysis identified a statistically significant relationship between P/L and protein (*p* = 0.010), with a good amount of variance explained by the model (R^2^ = 0.59), consistently with an earlier work [26]. This is supported by the results of MOLS analysis which found the same relationship between L and protein; as a result, the decrease in L and the increase in P during storage led to the inevitable increase in P/L values, as shown in Table 3. In conclusion, MOLS analysis did not find any statistically significant relationship with the environmental storage parameters.

### 3.3. Biscuit Characteristics 

Table 4 summarizes the results of the effects of storage time on biscuit volume and height.

**Figure 2:**
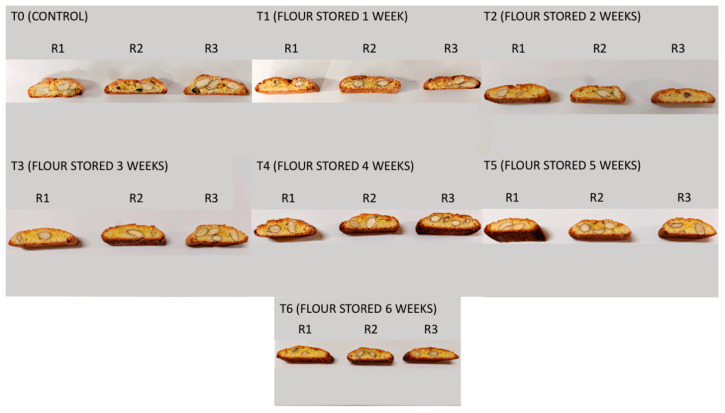
Biscuits (biscotto di Prato) obtained with the tested flours.

Figure 2 shows the biscuits produced during trials.

With respect to biscuit volume, the one-way ANOVA found a significant main effect for the factor storage time (*p* < 0.001). As shown in Table 4, the sample T0 had a significantly higher volume compared to all the other samples. MOLS analysis confirmed the results of the one-way ANOVA, highlighting a statistically significant relationship between biscuit volume and starch (*p* = 0.003), with a high amount of variance explained by the model (R^2^ = 0.83).

As highlighted by Ma & Baik (2018) [34], starch and damaged starch content are negatively correlated with biscuit volume. In particular, higher starch and damaged starch content increase flour water-holding capacity [28,34]. As a result, flours with high starch and damaged starch content are able to retain more water and the resulted biscuits had decreased water evaporation during baking, which resulted in biscuits of increased weight, and, consequently, lower volume [34]. In conclusion, as highlighted by Ma and Baik (2018) [34], starch and damaged starch content negatively affected biscuit volume by increasing the water retention capacity of the flour and biscuit weight.

In addition, MOLS analysis identified a statistically significant relationship between biscuit volume and weekly mean humidity (*p* = 0.006), with a low amount of variance explained by the model (R^2^ = 0.36). This is consistent with the findings for the alveograph parameter P (Section 3.2); however, given the higher amount of variance explained by the model biscuit volume-compositional parameters (i.e., R^2^ = 0.83), the changes in biscuit volume seems to be mainly related to flour composition (in particular to starch content). Regarding biscuit height, the one-way ANOVA did not find any statistically significant differences. Moreover, MOLS analysis did not find any relationship both with the compositional parameters of flour (moisture, starch, and protein) and with the environmental storage parameters (weekly mean temperature and humidity).

## 4. Conclusions

The results presented in this paper show the statistically significant effects of storage time on dough rheology and biscuit characteristics. Moreover, MOLS analysis highlighted that dough rheology and biscuit characteristics are mainly affected by flour composition (in particular from starch content), rather than from environmental storage parameters, in this short storage time. These results are supported by Shahid et al. (2019) [27] who found strong correlation between flour physiochemical attributes and dough rheology.

With respect to storage time, it significantly increased dough tenacity (P) and curve configuration ratio (P/L) which were optimal at T0 (control). However, 2–3 weeks of storage highlighted a significant increase in dough strength (W), an essential alveograph parameter that is closely correlated to the technological success of leavened products. This optimum found for dough strength (W) might be considered as a great stride in understanding the effects of storage time, confirming that wheat flour reaches its optimal performances after two–three weeks of storage, in particular for W. Moreover, this information could be useful not only for biscuits production, but especially for bread and bakery products (so for the entire bakery industry).

Additionally, storage time significantly affected the biscotto di Prato characteristics, highlighting the higher volume in the sample T0. However, this paper provides an additional step forward in understanding the effects of environmental storage conditions; in particular, the models developed using the MOLS analysis highlighted that dough rheology and biscuit characteristics are mainly affected by flour composition (in particular from starch content) rather than from environmental storage parameters, in the short storage period investigated. This short storage period was chosen since it reflects the approach of the majority of bread and biscuit factories which store the flours for a maximum of 6–7 weeks. However, despite a lower amount of variance explained by the models, weekly mean humidity was closely correlated with dough tenacity (P) and biscuit volume, highlighting the importance of this parameter during storage.

In conclusion, the results presented here should encourage the bakery industry to carefully analyze the composition of the flours used for the production of their products to optimize the biscuit characteristics (especially volume), as it is necessary to use flours with a low amount of damaged starch, as highlighted by Ma & Baik (2018) [34], and with a good protein content. This aim could be achieved selecting the most suitable milling technique and carefully managing the mill operative parameters, as reported in earlier works [4,6].

## Figures and Tables

**Table 1:** Results of flours characterization and analyses (based on dry weight). Results are expressed as the mean of the three replicates ± SD.

Sample	Moisture (g/100 g)	Starch (g/100 g)	Protein (g/100 g)
Flour T0 (control)	14.60 ± 0.19	65.33 ± 1.18	13.20 ± 0.25
Flour T1 (1 week of storage)	15.40 ± 0.21	68.26 ± 1.01	10.90 ± 0.49
Flour T2 (2 weeks of storage)	15.10 ± 0.16	68.97 ± 1.34	11.60 ± 0.36
Flour T3 (3 weeks of storage)	15.10 ± 0.19	69.70 ± 1.51	11.50 ± 0.41
Flour T4 (4 weeks of storage)	15.00 ± 0.11	68.60 ± 1.04	11.10 ± 0.53
Flour T5 (5 weeks of storage)	14.80 ± 0.16	69.10 ± 1.58	12.10 ± 0.46
Flour T6 (6 weeks of storage)	14.60 ± 0.22	70.90 ± 2.16	12.30 ± 0.38

**Table 2:** Weekly mean temperature and humidity in the company warehouse. Results are expressed as the mean of the three replicates ± SD.

Week	Mean Temperature (°C)	Mean Humidity (%)
Week 0 (control)	19.73 ± 0.29	64.33 ± 1.15
Week 1	19.88 ± 0.38	57.11 ± 3.01
Week 2	20.82 ± 0.59	58.00 ± 1.33
Week 3	20.47 ± 0.39	56.56 ± 3.15
Week 4	21.53 ± 0.43	55.44 ± 3.53
Week 5	23.04 ± 0.81	50.00 ± 1.45
Week 6	24.67 ± 0.29	54.33 ± 3.67

**Table 3:** Results of alveograph tests (mean of five measurements (diskettes) for each proof) expressed as the mean of the three replicates ± SD.

	P (Dough Tenacity)	L (Dough Extensibility)	G (Index of Swelling)	W (Deformation Energy)	P/L (Curve Configuration Ratio)
Flour T0 (control)	80.73 ± 1.62	74.73 ± 11.30	19.35 ± 1.37	237.87 ± 18.13	1.09 ± 0.16
Flour T1 (1 week of storage)	103.53 ± 1.97	63.07 ± 5.66	17.66 ± 0.80	269.53 ± 15.70	1.67 ± 0.17
Flour T2 (2 weeks of storage)	98.17 ± 3.88	72.12 ± 6.18	18.85 ± 0.80	282.37 ± 7.50	1.40 ± 0.16
Flour T3 (3 weeks of storage)	97.98 ± 5.55	69.07 ± 11.23	18.04 ± 2.16	295.63 ± 24.09	1.46 ± 0.18
Flour T4 (4 weeks of storage)	98.53 ± 3.26	58.60 ± 2.03	16.82 ± 0.42	242.05 ± 12.60	1.69 ± 0.02
Flour T5 (5 weeks of storage)	97.67 ± 1.22	60.93 ± 3.38	17.26 ± 0.55	248.47 ± 11.87	1.63 ± 0.13
Flour T6 (6 weeks of storage)	93.27 ± 2.16	59.87 ± 5.68	17.16 ± 0.78	237.47 ± 19.16	1.59 ± 0.15

**Table 4:** Results of biscuit characterization expressed as the mean of the three replicates ± SD.

Sample	Volume (L)	Height (mm)
Biscuit T0 (control)	0.0446 ± 0.003	17.47 ± 2.09
Biscuit T1 (1 week of flour storage)	0.0150 ± 0.006	15.37 ± 2.08
Biscuit T2 (2 weeks of flour storage)	0.0189 ± 0.004	16.07 ± 0.73
Biscuit T3 (3 weeks of flour storage)	0.0166 ± 0.003	17.58 ± 0.85
Biscuit T4 (4 weeks of flour storage)	0.0191 ± 0.001	18.38 ± 0.98
Biscuit T5 (5 weeks of flour storage)	0.0170 ± 0.002	17.58 ± 2.09
Biscuit T6 (6 weeks of flour storage)	0.0272 ± 0.002	16.88 ± 0.90

## Data Availability

The datasets generated for this study are available on request to the corresponding author.
